# Explainability Challenges in Medical AI: The Conceptual Hidden Cost of Machine Learning Preprocessing

**DOI:** 10.1002/hsr2.71776

**Published:** 2026-04-13

**Authors:** Ahmed M. Salih

**Affiliations:** ^1^ Department of Population Health Sciences University of Leicester Leicester UK; ^2^ William Harvey Research Institute, NIHR Barts Biomedical Research Centre Queen Mary University of London London UK; ^3^ Barts Heart Centre St Bartholomew's Hospital, Barts Health NHS Trust London UK; ^4^ PRIME Lab, Scientific Research Center University of Zakho Kurdistan Region Iraq

**Keywords:** Medicine, preprocessing, XAI

## Abstract

**Background:**

Data preprocessing is a significant step in machine learning to improve the performance of the model and decreases the running time. This might include dealing with missing values, outliers' removal, data augmentation, dimensionality reduction and handling the confounding variables.

**Objective:**

This commentary explores the common preprocessing steps used in medical machine learning and highlights their conceptual hidden cost including reduced model explainability and clinical interpretability. This commentary focuses on conceptual rather than empirical implications of these preprocessing decisions.

**Methods:**

Literature review was undertaken to explore the data preprocessing steps in machine learning.

**Results:**

Although it is found the preprocessing steps improve the accuracy of the model, but they might block new findings and hinder the explainability of the model if they are not carefully considered especially in medicine. We identify key risks such as bias introduction and over simplification and outline mitigation strategies.

**Conclusion:**

The novelty of this work lies in systematically connecting preprocessing practices with explainability challenges in healthcare artificial intelligence and suggesting approaches to balance performance with explainability. This highlights the need for careful design of preprocessing pipelines in medical artificial intelligence systems to ensure both reliable predictions and clinical trust.

## 1 Introduction

Data preprocessing is an initial indispensable step in machine learning and data science to improve the performance of the model and decreases the running time. It consists of several components and processing steps that are performed on raw data to ensure its quality before fitting them to any machine learning model [1]. On the other hand, explainable artificial intelligence (XAI) as an emerging topic aims to understand how a machine learning model works. Its direct aims are more to do with improving the explainability of the model than improving its performance [2]. Interpretability on the other hand is to what extent the internal mechanics of a model is understandable from human point of view. Several XAI methods were developed and proposed to explain different models and data type including SHapley Additive exPlanations (SHAP) [3] and Local Interpretable Model‐Agnostic Explanations (LIME) [4] are mostly used with tabular data while heatmap‐based XAI methods including Gradient‐weighted Class Activation Mapping (Grad‐CAM) are used for imaging data [5]. Data preprocessing and XAI should not hinder each other. They should rather work together to improve the performance of the model and its explainability simultaneously. In other words, any step or steps to improve the performance of the model should not affect its explainability negatively. However, current approaches of data‐preprocessing might hinder the aims of the XAI and eventually its explainability and interpretability.

This paper discusses some essential steps of data preprocessing in machine learning and how they might improve the accuracy of the model but hinder its explainability.

## 2 Data Preprocessing Steps

**Figure 1:**
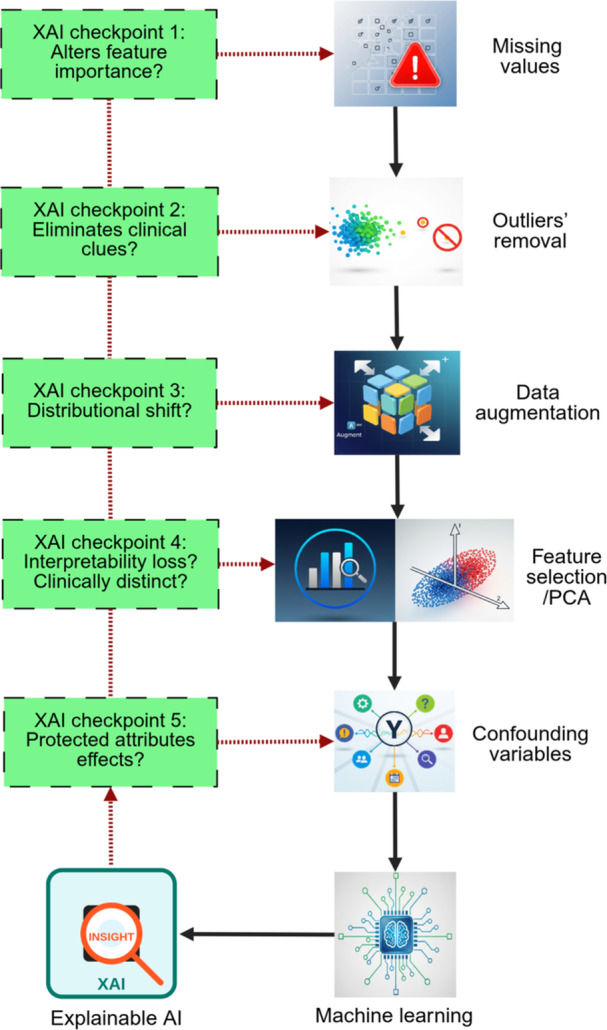
Conceptual framework for integrating explainability into data preprocessing.

Figure 1 illustrates the conceptual framework for integrating explainability into data preprocessing that are commonly applicable across diverse types of data. The subsequent subsection examines each of these steps in detail, with particular emphasis on their associated challenges for XAI.

### 2.1 Missing Values

Missing values is one the most prevalent issue in machine learning especially in medicine which might impair the explainability of the model. Usually, the data are not complete for all individuals for variety of reasons. Possible way to deal with the missing values is to remove them either at individuals' level or at features level when the missing rate is high based on a predetermined threshold or impute them [6]. XAI and interpretability is significantly affected by the chosen method to impute the missing values [7]. Moreover, as the real value is unknown, using those imputation methods might lead to a case which is called counterfactuals [8]. Two studies [7, 8] discussed how the imputation method might alter the explanation and consequently might have negative impact on the human well‐being and safety. It is vital to use more than one imputation method with several models and XAI to compare and contrast how the explanation will be different.

### 2.2 Outliers' Removal

One of the most common steps in machine learning modeling is outliers' removal. There are multiple methods to identify the outliers including those based on statistical methods [9]. However, aligning with the aims of the XAI, every data point which represent valuable information of an individual need to be explained and should not be ignored. Clinically speaking, outliers might hold a clinical interpretation which explain why the values are very big or small. They might represent a new case that has not be investigated before. For instance, in cardiology, outlier detection methods might identify an unusual ECG signal that holds implications for rare arrhythmias [10]. XAI should be used to explain and interpret why the model detected those data points as outliers and why their values are deviated from the norm values. Possible solution could be by implementing clustering models where those with extreme values are combined together and modeled separately. Other way could by applying some XAI methods where first the outliers are detected and then explained why they hold such extreme values [11]. Finally, it is vital to compare the outcome of XAI before and after applying outliers' removal methods if such methods are deemed necessary.

### 2.3 Data Augmentation

In medicine it is common that the number of cases and control are not similar. This is an issue when applying a classification model which is called imbalanced data. The possible solution in this case is the data augmentation to increase the number of cases or control [12]. Data augmentation should be implemented and interpreted accurately. Considering a case where the number of cases is massively smaller than the number of controls for a given population (Alzheimer's disease vs control). If data augmentation is performed on all data set, then the data set does not represent the population anymore neither the generated explanation can be generalized over the population. On the other hand. if the data augmentation is performed only on the training data, then the test and the training data will have different distribution and the model performance might be poor on the test data. Consequently, different explanation might be obtained when XAI is applied to training and/or test data. Possible solution could be through applying several data augmentation methods and examine how the explanation will be different compared with a baseline. Moreover, it is more reasonable to apply XAI methods where data augmentation is embedded in the model [13].

### 2.4 Feature Selection

One of the most common steps in machine learning modeling is feature selection. It is especially recommended when the features are collinear, some of them redundant or irrelevant which might decrease the running time and improve model performance [14]. Feature selection might prevent or block some clinical explanation. For instance, glucose and insulin are found to be associated positively in the blood in a non‐diabetic individual [15]. Other example could the association of ApoE4 with t‐tau protein. It has been found that there is a significant association between the two in individuals with Alzheimer's disease and dementia [16]. Similarly, HER2 and estrogen receptor are two proteins over expressed in breast cancer patients [17]. Feature selection might pick one of them and consider the other as a redundant feature because they are highly correlated. However, the clinical interpretation of each one of them might be different when they appear among the most informative features toward the outcome. Feature selection should not be based only on the statistical criterion. Clinical information might also be used to select the features.

### 2.5 Principal Component Analysis

Principal component analysis (PCA) is one of the most common method used in machine learning as a dimensionality reduction technique [18]. It converts the raw data into uncorrelated principal components while preserving the original data's information and variability as much as possible. The implementation of PCA hinders achieving the principles of XAI for several reasons. Firstly, applying PCA means converting the new PCs into unitless. After applying an XAI method, it is really not clear how to interpret the outcome of XAI in units. Secondly, it is challenging to even identifying the most informative features in the model after they have been transferred into PCs. Some might argue that the new components might be returned into their original space and accordingly will be able to identify the most informative features. However, the contribution of each feature is allocated into several components and it is challenging to be precise how exactly they contribute in the model outcome. PCA might be less appropriate in medicine because of the reasons mentioned before.

### 2.6 Confounding Variables

In most of the clinical researches there are confounding variables. They might be at individual levels such as sex, ethnicity, age, and weight or they could be related to data acquisition [19]. Dealing with the confounding variables might have significant impact on the explainability of the model. For instance, one of the approaches to mitigate the impact of the confounding variables is by applying a matching method (e.g., propensity score) where cases and control are matched based on sets of confounding variables. Although this method might reduce the impact of the confounding variables, however it might block a clinical clue which might be captured by XAI methods. For example, it is well established of sex differences in the risk of cardiovascular [20] and neurodegenerative diseases [21]. Moreover, ethnic minority groups including Black are more at risk of infection of COVID‐19 than White ethnicity [22]. When applying the matching method using confounding variables (ethnicity and sex among them), XAI cannot capture of the ethnicity and sex differences in these two conditions. Other approach is to include them in the model alongside the independent variables. While this would help to explain the model when switching between their values (male to female, or between ethnic groups), however the classic interpretation (holding other variables as constant) of the coefficient value in the model makes it difficult to understand.

### 2.7 Performance/XAI Trade‐Offs

While the inherent trade‐off between model performance (accuracy) and explainability (interpretability) is widely explored in medical machine learning literature, these discussions often focus on the model architecture itself (e.g., deep neural networks *vs.* linear models). Crucially, the systemic erosion of interpretability caused by the earliest, often unexamined, preprocessing steps is frequently overlooked in this context. To ground our conceptual analysis in real‐world challenges, we note that established benchmarks like the Medical Information Mart for Intensive Care (MIMICIII) [23] dataset and the Alzheimer's Disease Neuroimaging Initiative (ADNI) [24] are routinely subjected to the aggressive preprocessing methods discussed in this paper (e.g., extensive imputation, outlier removal, and dimensionality reduction). This common practice means that the performance and explainability results reported on these critical datasets are often based on transformed data that bears little resemblance to the original clinical reality. Our proposed conceptual framework, introducing XAI checkpoints within the preprocessing pipeline, is therefore highly relevant to future empirical research aiming to achieve a more trustworthy balance of performance and true clinical interpretability on these standard benchmarks.

## 3 Conclusion

To conclude, Table 1 summarizes the aforementioned steps, related risks and possible XAI solutions. There is no a magic solution and each method or approach to adopt to improve the model explainability has its own strengths and weaknesses. The case of electronic health records illustrates how rapid technological adoption without adequate attention to data integrity, interoperability, and clinical context can lead to systemic failures, including clinician burnout and compromised patient safety [25]. This underscores the importance of aligning preprocessing strategies with the principles of XAI, especially in sensitive domains like medicine. Furthermore, the impact of preprocessing steps on explainability should be evaluated not only in isolated scenarios but also across diverse benchmarks and datasets. Benchmarking against widely used datasets such as Medical Information Mart for Intensive Care for critical care or Alzheimer's Disease Neuroimaging Initiative for neurodegenerative diseases can help reveal how preprocessing choices influence model explainability and interpretability in different clinical contexts. This comparative lens is essential for developing generalizable and trustworthy AI systems in healthcare. Future AI systems must be designed not only for accuracy but also for transparency, fairness, and clinical relevance to ensure trust and safety in real‐world applications. Future work will empirically test these conceptual insights using benchmark datasets such as MIMIC‐III and ADNI to evaluate the trade‐offs between performance and explainability.

**Table 1:** Conceptual XAI workflow for mitigating preprocessing risks in medical machine learning.

Steps	Risks	Possible XAI solutions
Missing values (Imputation)	Introduces counterfactuals and alters feature distributions, leading to inconsistent or incorrect explanations of original data.	Compare and contrast explanations derived from multiple imputation methods against a baseline; ensure stability of explanations across imputation strategies.
Outliers' removal	Ignores potentially critical clinical cases (rare events, novel conditions) that hold high interpretive value, leading to biased model explanations.	Use XAI methods to explain the outlier itself (why it has an extreme value) before removal; model combined outlier groups separately using clustering.
Data augmentation	Creates a synthetic dataset that no longer represents the true population distribution, rendering explanations non‐generalizable, especially when applied globally.	Apply augmentation methods selectively (e.g., only on training data) and rigorously compare XAI results on training versus original test sets to understand distributional shift.
Feature selection	Blocks redundant features that are highly correlated but have distinct, essential clinical interpretations (e.g., glucose/insulin, ApoE4/t‐tau), leading to incomplete feature importance maps.	Incorporate clinical knowledge alongside statistical criteria for feature selection; compare XAI results (e.g., feature importance scores) between reduced and full feature sets.
PCA	Loss of feature semantics and unit clarity; conversion of raw features into unitless PCs makes XAI outputs (e.g., impact of a PC) clinically uninterpretable.	Avoid PCA in clinical domains where interpretability is paramount; if used, rigorously attempt to map PC contributions back to the original feature space for interpretation.
Confounding variables (Matching)	Matching methods (e.g., propensity score) systematically hide or block established clinical differences (e.g., sex/ethnicity risks), preventing XAI from capturing these critical insights.	Include confounders directly in the model as independent variables; use XAI (e.g., counterfactual explanations) to explore impact by systematically switching confounder values (e.g., male to female).

## Author Contributions

**Ahmed M. Salih:** conceptualization, writing – original draft, editing and finalized the manuscript.

## Ethics Statement

The author has nothing to report.

## Consent

The author has nothing to report.

## Conflicts of Interest

The author declares no conflicts of interest.

## Transparency Statement

The lead author Ahmed M. Salih affirms that this manuscript is an honest, accurate, and transparent account of the study being reported; that no important aspects of the study have been omitted; and that any discrepancies from the study as planned (and, if relevant, registered) have been explained.

## Data Availability

Data sharing not applicable to this article as no data sets were generated or analyzed during the current study.
